# Interleukin-6/interleukin-6 receptor complex promotes osteogenic differentiation of bone marrow-derived mesenchymal stem cells

**DOI:** 10.1186/s13287-017-0766-0

**Published:** 2018-01-22

**Authors:** Zhongyu Xie, Su’an Tang, Guiwen Ye, Peng Wang, Jinteng Li, Wenjie Liu, Ming Li, Shan Wang, Xiaohua Wu, Shuizhong Cen, Guan Zheng, Mengjun Ma, Yanfeng Wu, Huiyong Shen

**Affiliations:** 1 Department of Orthopedics, Sun Yat-sen Memorial Hospital, Sun Yat-sen University, 107# Yan Jiang Road West, Guangzhou, Guangdong 510120 People’s Republic of China; 2 Center for Biotherapy, Sun Yat-sen Memorial Hospital, Sun Yat-sen University, 107# Yan Jiang Road West, Guangzhou, Guangdong 510120 People’s Republic of China

**Keywords:** Bone marrow-derived mesenchymal stem cells, Osteogenic differentiation, Interleukin-6, Interleukin-6 receptor

## Abstract

**Background:**

Interleukin-6 (IL-6) with IL-6 receptor (IL-6R) play an important role in the tissue regeneration in vivo, especially bone metabolism. Bone marrow -derived mesenchymal stem cells (BM-MSCs) are multipotent stromal cells, which are main origin of osteoblasts. However, the roles of IL-6 and IL-6R in the osteogenic differentiation of BM-MSCs are still unclear.

**Methods:**

The expression of IL-6 and IL-6R was detected in BM-MSCs during osteogenic differentiation. The activation of the STAT3 pathway was assessed and its role in the osteogenic differentiation of BM-MSCs was determined using the specific inhibitor AG490. Exogenous IL-6/soluble IL-6R or antibodies against IL-6/IL-6R were used to confirm the mechanism by which the IL-6/IL-6R complex promotes the osteogenic differentiation.

**Results:**

The levels of IL-6 and IL-6R, especially the level of membranous IL-6R but not that of soluble IL-6R, increased during osteogenic differentiation in BM-MSCs. The levels of IL-6 and IL-6R were positively correlated with the osteogenic potential of BM-MSCs. The STAT3 signaling pathway was activated during the osteogenic differentiation of BM-MSCs. AG490 markedly inhibited the activation of the STAT3 pathway and, subsequently, the osteogenic differentiation potential of BM-MSCs. Additionally, exogenous IL-6 and soluble IL-6R accelerated the osteogenic differentiation of BM-MSCs. In contrast, antibodies against IL-6 or IL-6R suppressed the osteogenic differentiation of BM-MSCs. Moreover, IL-6 and IL-6R were found to stimulate each other’s expression in BM-MSCs.

**Conclusions:**

IL-6 and IL-6R levels increase during the osteogenic differentiation of BM-MSCs. These two molecules form a complex to activate the downstream STAT3 signaling pathway, which promotes osteogenic differentiation in BM-MSCs via an autocrine/paracrine feedback loop.

**Electronic supplementary material:**

The online version of this article (10.1186/s13287-017-0766-0) contains supplementary material, which is available to authorized users.

## Background

Mesenchymal stem cells (MSCs) are multipotent stromal cells that are primarily located in connective tissues, especially in the bone marrow [1]. Defined as multipotent stromal cells, bone marrow-derived mesenchymal stem cells (BM-MSCs) are characterized by low immunogenicity and powerful immunosuppressive activity [2]. BM-MSCs are capable of trilineage differentiation into osteoblasts, chondroblasts or adipoblasts [3]. This multipotency of BM-MSCs enable their use as seed cells in numerous clinical applications, such as tissue engineering [4]. However, the differentiation potential of BM-MSCs is profoundly affected by various factors in vivo, and the associated mechanisms have not yet been elucidated.

Interleukin-6 (IL-6) is a pleiotropic cytokine that plays an important role in immune regulation, hematopoiesis and tissue regeneration in vivo [5]. IL-6 binds to IL-6 receptor (IL-6R) and gp130, which mainly activates the STAT3 signaling pathway downstream and promotes gene transcription [5]. BM-MSCs both secrete and respond to IL-6 [6]. A previous study demonstrated that autocrine/paracrine IL-6 contributes to the chondrogenic differentiation of BM-MSCs [7]. However, the effect of autocrine/paracrine IL-6 in the osteogenic differentiation of BM-MSCs is still controversial.

IL-6R has two isoforms, namely, membrane-bound IL-6R (mIL-6R) and soluble IL-6R (sIL-6R) [8]. The latter is either produced by the proteolytic cleavage of mIL-6R or is directly translated from alternatively spliced mRNA. In contrast to most other soluble receptors, mIL-6R and sIL-6R exert the same effect. sIL-6R can bind to the ligand IL-6 with comparable affinity to that of mIL-6R, which activates the downstream STAT3 signaling pathway and acts as an IL-6 agonist [9]. It is known that IL-6 affects the function of BM-MSCs through an autocrine/paracrine feedback loop by binding to IL-6R [7]. However, the role of IL-6R in the osteogenic differentiation of BM-MSCs needs to be further investigated.

To explore the questions mentioned above, we determined the expression and function of the IL-6/IL-6R complex during the osteogenic differentiation of BM-MSCs. We found that under osteogenic induction, BM-MSCs continuously secreted IL-6, which formed a complex with mIL-6R and finally promoted the osteogenic differentiation of these cells. We speculated that the IL-6/mIL-6R complex may have important clinical potential in the future.

## Methods

### Cell isolation and culture

This study was approved by the ethics committee of Sun Yat-Sen Memorial Hospital, Sun Yat-Sen University, Guangzhou, China. Fifteen healthy donors were recruited into the study. The age of the healthy donors was 29.1 ± 7.6 years and eight were male (53.3%). The donors signed informed consent after being informed of the possible risks. Bone marrow aspirations were performed by skilled doctors according to internationally accepted standardized procedures. The bone marrow samples were immediately processed, and BM-MSCs were isolated using a density gradient centrifugation method as previously described [10]. BM-MSCs were cultured in Dulbecco’s modified Eagle’s medium (DMEM, Gibco, Waltham, MA, USA) with 10% fetal bovine serum (FBS, Sijiqing Biological Engineering Material Company Limited, Hangzhou, PR China) at 37 °C/5% CO_2_. Medium was replaced every 3 days, and BM-MSCs were passaged when the culture reached approximately 90% confluency. The cells were used for experiments at passage 3.

### Flow cytometry

For phenotype identification, BM-MSCs were digested with 0.25% trypsin containing 0.53 mM EDTA and washed with phosphate-buffered saline (PBS). After incubation with antibodies against CD29, CD73, CD90, CD105, CD14, CD34, CD45, and HLA-DR (BD Pharmingen, Franklin Lakes, NJ, USA), the phenotype of BM-MSCs was detected using a BD Influx cell sorter (BD Biosciences, San Jose, CA, USA). For the detection of mIL-6R, BM-MSCs were incubated with anti-human IL-6R antibody (R&D Systems, Minneapolis, MN, USA), and mIL-6R^+^ cells were detected by flow cytometry.

### Trilineage differentiation potential assay

For osteogenic differentiation, BM-MSCs were seeded in 12-well plates at a density of 1.5 × 10^4^ cells/cm^2^ and cultured in osteogenic differentiation medium containing DMEM with 10% FBS, 100 IU/ml penicillin, 100 IU/ml streptomycin, 0.1 μM dexamethasone, 10 mM β-glycerol phosphate and 50 μM ascorbic acid (Sigma-Aldrich, St. Louis, MO, USA). The medium was replaced every 3 days. Osteogenic differentiation potential of these cells was determined by Alizarin Red S (ARS) staining as described below on day 21 of induction.

For chondrogenic differentiation, BM-MSCs were centrifuged at 600 g for 5 minutes to form pellets. The cells were then cultured in chondrogenic differentiation medium containing 100 IU/ml penicillin, 100 IU/ml streptomycin, 1% ITS-Premix (Corning, Corning, NY, USA), 50 μM ascorbic acid (Sigma-Aldrich), 1 mM sodium pyruvate (Sigma-Aldrich), 0.1 μM dexamethasone, and 10 ng/mL transforming growth factor-β3 (R&D Systems). The medium was replaced every 3 days. On day 21 of induction, cell pellets were fixed with 4% paraformaldehyde, embedded in paraffin, and subjected to Alcian Blue staining to determine their chondrogenic differentiation potential.

For adipogenic differentiation, BM-MSCs were seeded in 12-well plates at a density of 1.5 × 10^4^ cells/cm^2^ and cultured in adipogenic differentiation medium containing DMEM with 10% FBS, 1 μM dexamethasone, 10 μg/ml insulin (Sigma-Aldrich), 0.5 mM 3-isobutyl-1-methylxanthine (Sigma-Aldrich) and 0.2 mM indomethacin (Sigma-Aldrich). After 3 days of induction, the medium was replaced with DMEM containing 10 μg/ml insulin, and 1 day later, the medium was replaced with induction medium. On day 21 of induction, BM-MSCs were fixed with 4% paraformaldehyde and subjected to Oil Red O staining to determine their adipogenic differentiation potential.

### Quantitative real-time PCR

Total RNA was extracted using TRIzol reagent (Life Technologies, Carlsbad, CA, USA) on the day 10 of induction. Synthesis of cDNA was performed using PrimeScript™ RT reagent kits (TaKaRa, Shiga, Japan). Quantitative real-time PCR was performed on a LightCycler® 480 PCR system (Roche, Basel, Switzerland) using SYBR® Premix Ex Taq™ kits (TaKaRa). The reactions conditions were 30 s at 95 °C, 40 cycles of 5 s at 95 °C and 20 s at 60 °C. The relative expression levels of each gene were analyzed using the 2^-ΔΔCt^ method and normalized to GAPDH expression. The sequences of forward and reverse primers for each gene are shown in Additional file 1: Table S1.

### Enzyme-linked immunosorbent assay (ELISA)

Cell culture supernatants were collected 24 h after the last medium change at the indicated time points. Protein level of IL-6 or sIL-6R in cell culture supernatants was detected using Human IL-6 and IL-6R Quantikine ELISA Kits (R&D Systems) according to the manufacturer’s instructions.

### Western blotting

MSCs were lysed using RIPA buffer (Sigma-Aldrich) containing protease and phosphatase inhibitors (Roche). The lysate was centrifuged at 4 °C for 30 min, and the protein concentration in the supernatant was measured using a BCA assay kit (Sigma-Aldrich). Equal concentrations of proteins were separated by 10% sodium dodecyl sulfate-polyacrylamide gel electrophoresis and then transferred to a polyvinylidene fluoride membrane (EMD Millipore Burlington, MA, USA), followed by overnight blotting with primary antibodies against GAPDH, IL-6R, STAT3 and phosphorylated-STAT3 (1:1000, Cell Signaling Technology Danvers, MA, USA). The membrane was then washed and incubated with horseradish peroxidase (HRP)-conjugated secondary antibody (1:3000, Cell Signaling Technology) for 1 h. Specific antibody-antigen complexes were detected using the Immobilon Western Chemiluminescent HRP Substrate (EMD Millipore).

### Alizarin Red S (ARS) staining and quantification

MSCs were fixed in 4% paraformaldehyde and then stained with 1% ARS (pH 4.3) for 15 min. After being washed three times, the stained cells were visualized under a microscope and photographed and then de-stained using 10% cetylpyridinium chloride monohydrate (CPC, Sigma-Aldrich). A 200-μl aliquot of the mixture was transferred to a 96-well plate, and the absorbance was measured at 562 nm.

### Alkaline phosphatase (ALP) activity and staining

Proteins were extracted as described above. ALP activity was detected using ALP activity kits (Nanjing Jiancheng Biotech, Nanjing, China) according the manufacturer’s protocol. ALP activity was expressed as units per gram protein per 15 min (U/gpro/15 min). For ALP staining, MSCs were fixed in a citrate-acetone-formaldehyde fixative and then treated with an alkaline dye (Sigma-Aldrich) for 15 min in the dark. The stained cells were visualized under a microscope and photographed.

### Exogenous stimulation assay

AG490, a specific STAT3 signaling pathway inhibitor, was added at a concentration of 10 μM during the osteogenic differentiation of MSCs. Recombinant human IL-6 protein (100 ng/ml), recombinant human sIL-6R protein (100 ng/ml), anti-IL-6 antibody (5 μg/ml) or anti-IL-6R antibody (5 μg/ml) was added to determine the roles of IL-6/IL-6R in the osteogenic differentiation of MSCs (all from R&D Systems).

### Lentiviruses construction and infection

Lentiviruses encoding short hairpin RNA (shRNA) for IL-6 were constructed with a target sequence of 5′- GCAGGACATGACAACTCATCT-3′ (Lv-IL6), and shRNA for IL-6R were also constructed with a target sequence of 5′- GGAAGACAATGCCACTGTTCA-3′ (Lv-IL6R). The sequence for the negative control shRNA was 5′-TTCTCCGAACGTGTCACGTTTC-3′ (Lv-NC). Lentiviruses were generated by co-transfecting pGLVH1/GFP/Puro (Gene Pharma, Shanghai, China) and packing plasmids (pGag/Pol, pRev, and pVSV-G) into 293 T cells. The culture supernatants containing lentiviruses were filtered and concentrated 72 hours after transfection. Lentiviruses (10^9^ TU/ml) with polybrene (5 μg/ml) were added into medium and incubated with MSCs for 24 h at a multiplicity of infection (MOI) of 50. Related experiments were performed on day 10 of induction.

### Statistical analysis

All the samples were detected by individual, and all the experiments were technically repeated three times. All data were expressed as the means ± standard deviations (SDs). *T* test and one-way analysis of variance followed by the Bonferroni test and Pearson correlation test were performed for statistical analyses using SPSS Version 13.0 (IBM SPSS Inc., Armonk, NY, USA). *P* values less than 0.05 were considered statistically significant.

## Results

### Phenotype identification and trilineage differentiation potential of BM-MSCs

**Fig. 1:**
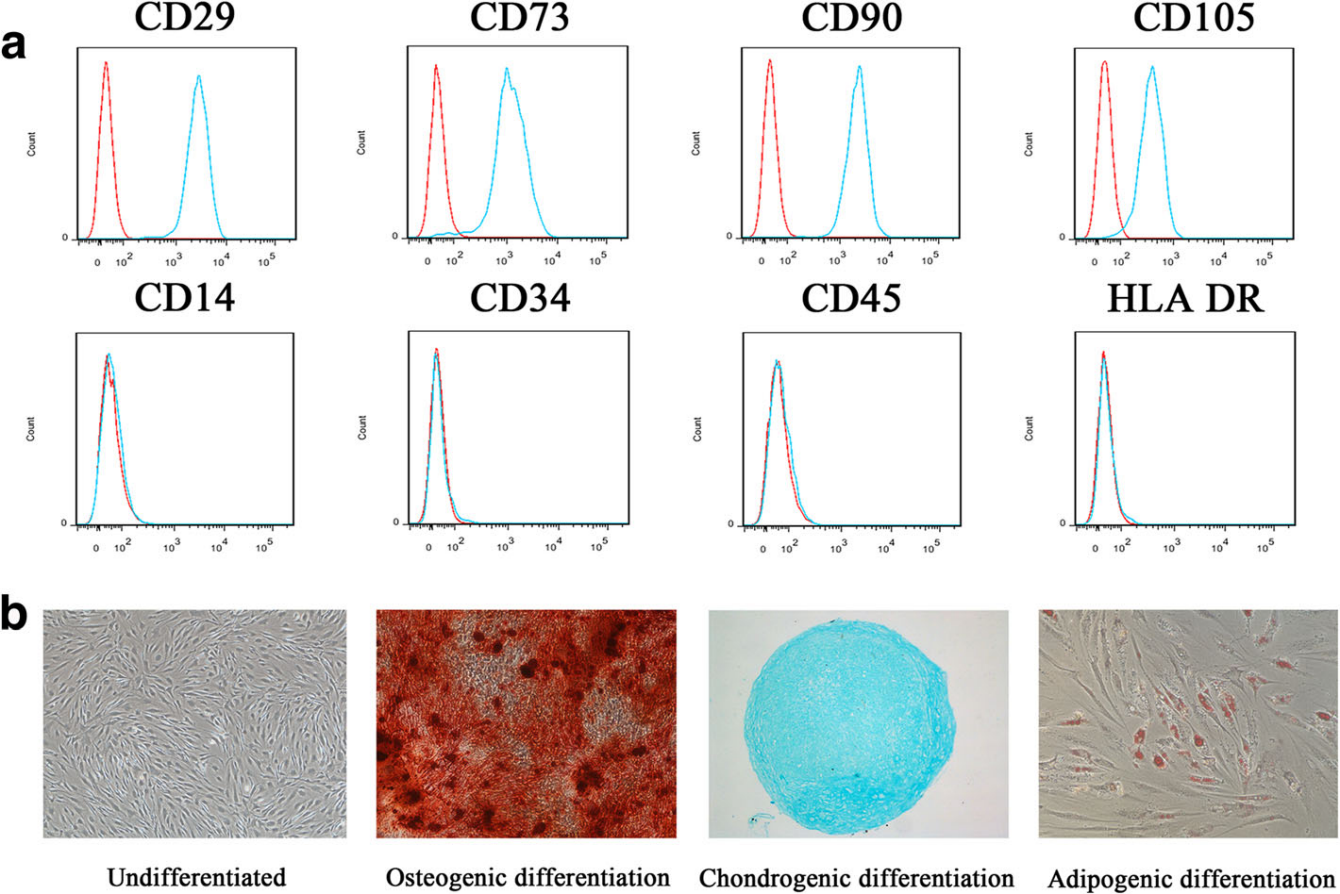
Phenotype identification and trilineage differentiation potential of BM-MSCs. **a** BM-MSCs were positive for CD29, CD73, CD90, and CD105 and negative for CD14, CD34, CD45, and HLA-DR. **b** BM-MSCs could be induced to undergo osteogenic differentiation, chondrogenic differentiation and adipogenic differentiation

To determine the purity and characteristics of BM-MSCs, we detected their phenotype using flow cytometry. The isolated BM-MSCs were positive for CD29, CD73, CD90, and CD105 and negative for CD14, CD34, CD45, and HLA-DR (Fig. 1a), characteristic of MSCs. To determine the multipotent differentiation potential of BM-MSCs, the cells were subjected to osteogenic, chondrogenic and adipogenic differentiation conditions. ARS staining confirmed the osteogenic differentiation of BM-MSCs. Alcian blue staining confirmed the chondrogenic differentiation of BM-MSCs. Finally, Oil Red O staining confirmed the adipogenic differentiation of BM-MSCs (Fig. 1b). Therefore, the BM-MSCs isolated for this study met the standard for MSCs specified by the International Society for Stem Cell Research (ISCT) [11].

### IL-6 and IL-6R expression in BM-MSCs during osteogenic differentiation

**Fig. 2:**
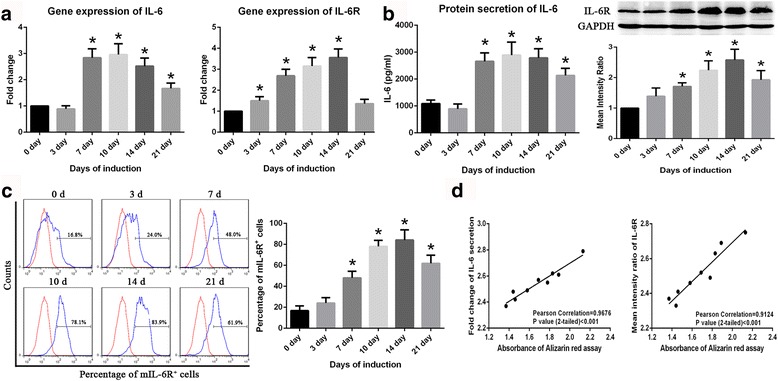
IL-6 and IL-6R expression in BM-MSCs during osteogenic differentiation. **a** Expression of IL-6 and IL-6R genes increased during osteogenic differentiation and peaked on day 10 or 14 of induction. **b** ELISA results showing that IL-6 secretion increases during osteogenic differentiation in BM-MSCs. Western blotting results showing that IL-6R expression in BM-MSCs peaked on day 14 of induction. **c** Results of flow cytometry showing that mIL-6R expression increases during osteogenic differentiation in BM-MSCs. **d** IL-6 and IL-6R expression are positively correlated with ARS staining results in BM-MSCs. Data are presented as the means ± SD of 15 samples per group. *Indicates *P* < 0.05 compared to the day 0 group. *IL-6* interleukin 6, *IL-6R* interleukin 6 receptor

To explore the role of IL-6 and IL-6R in the osteogenic differentiation of MSCs, we first detected the expression levels of these two factors during osteogenic differentiation. With the progression of osteogenic differentiation, the mRNA levels of IL-6 and IL-6R gradually increased and peaked on day 10 or 14 of induction (Fig. 2a). The levels of IL-6 and IL-6R protein exhibited the same trend (Fig. 2b). IL-6R exists in two different forms, namely, mIL-6R and sIL-6R. To our surprise, during osteogenic differentiation, the BM-MSCs did not secrete sIL-6R (data from ELISA not shown). Based on the results of flow cytometry, the expression of mIL-6R and peaked on day 14 of induction, which was consistent with the expression of total IL-6R protein (Fig. 2c). In addition, the levels of both IL-6 and IL-6R were positively correlated with the results of ARS staining, which is used to detect osteogenic differentiation in BM-MSCs, indicating a relationship between IL-6/IL-6R expression levels and the osteogenic differentiation potential of BM-MSCs (Fig. 2d).

### Activation of the STAT3 signaling pathway during osteogenic differentiation in BM-MSCs

**Fig. 3:**
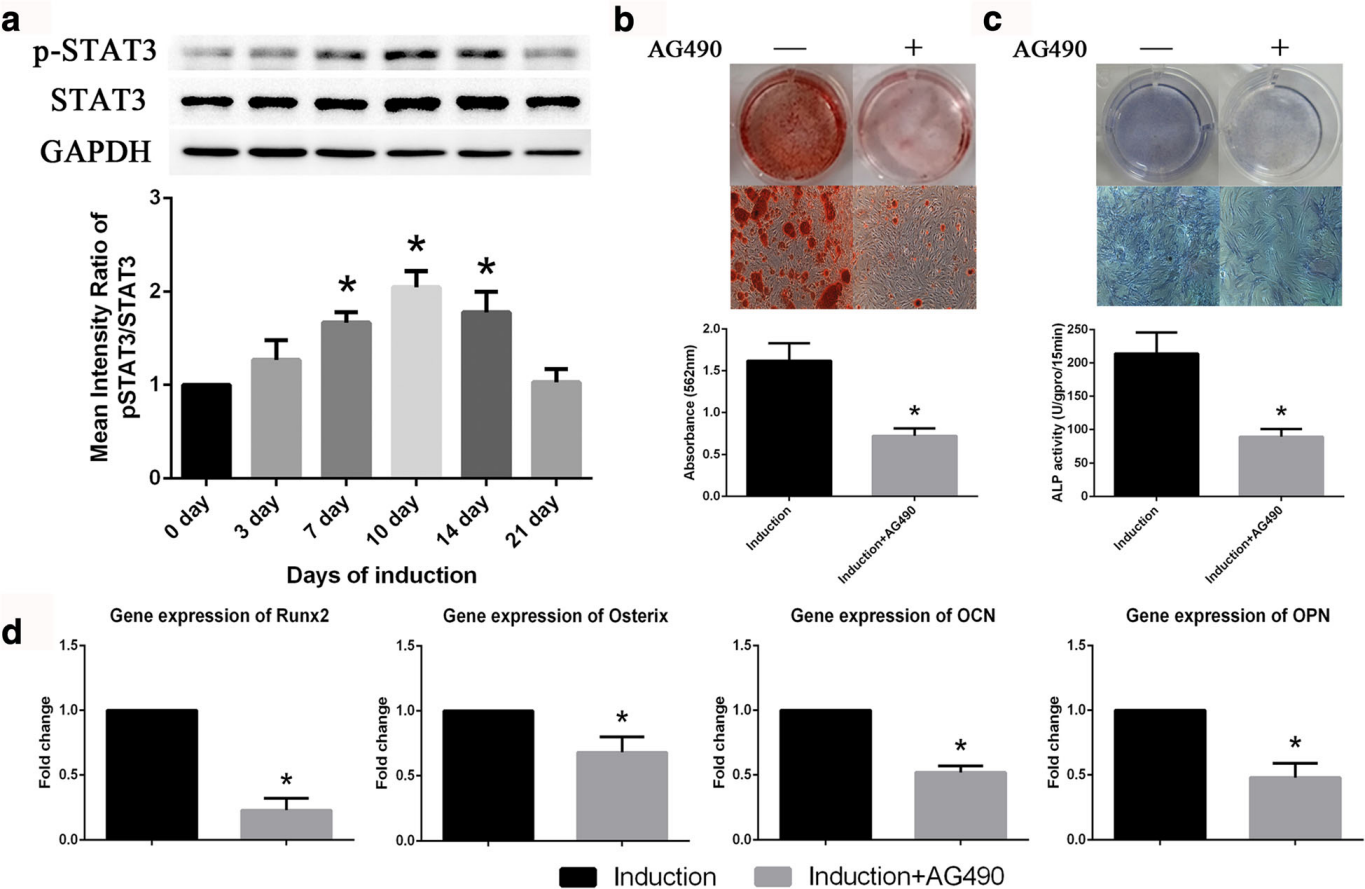
Activation of the STAT3 signaling pathway during osteogenic differentiation in BM-MSCs. **a** STAT3 phosphorylation was markedly increased on day 7 to 14 of induction. **b** Extent of ARS staining in BM-MSCs on day 10 of osteogenic differentiation was reduced by AG490. **c** ALP staining and activity of BM-MSCs on day 10 of osteogenic differentiation were reduced by AG490. **d** Expression of osteoblastic markers in BM-MSCs, including Runx2, Osterix, OCN, and OPN, was inhibited by AG490. Data are presented as the means ± SD of 15 samples per group. *Indicates *P* < 0.05 compared to the day 0 group. *IL-6* interleukin 6, *IL-6R* interleukin 6 receptor, *OCN* osteocalcin, *OPN* osteopontin

IL-6 binds to IL-6R and activates the STAT3 signaling pathway [5]. During osteogenic differentiation, STAT3 phosphorylation was increased, reached the highest level on day 10 of induction and then decreased (Fig. 3a), which was consistent with the expression profiles of IL-6 and IL-6R. AG490, a specific inhibitor of the STAT3 pathway, markedly inhibited the osteogenic differentiation of BM-MSCs as shown by ARS and ALP staining (Fig. 3b, c). Blocking the STAT3 pathway also inhibited the expression of osteoblastic marker genes, including Runx2, Osterix, osteocalcin (OCN) and osteopontin (OPN) (Fig. 3d).

### Exogenous IL-6 and sIL-6R promote osteogenic differentiation in BM-MSCs

**Fig. 4:**
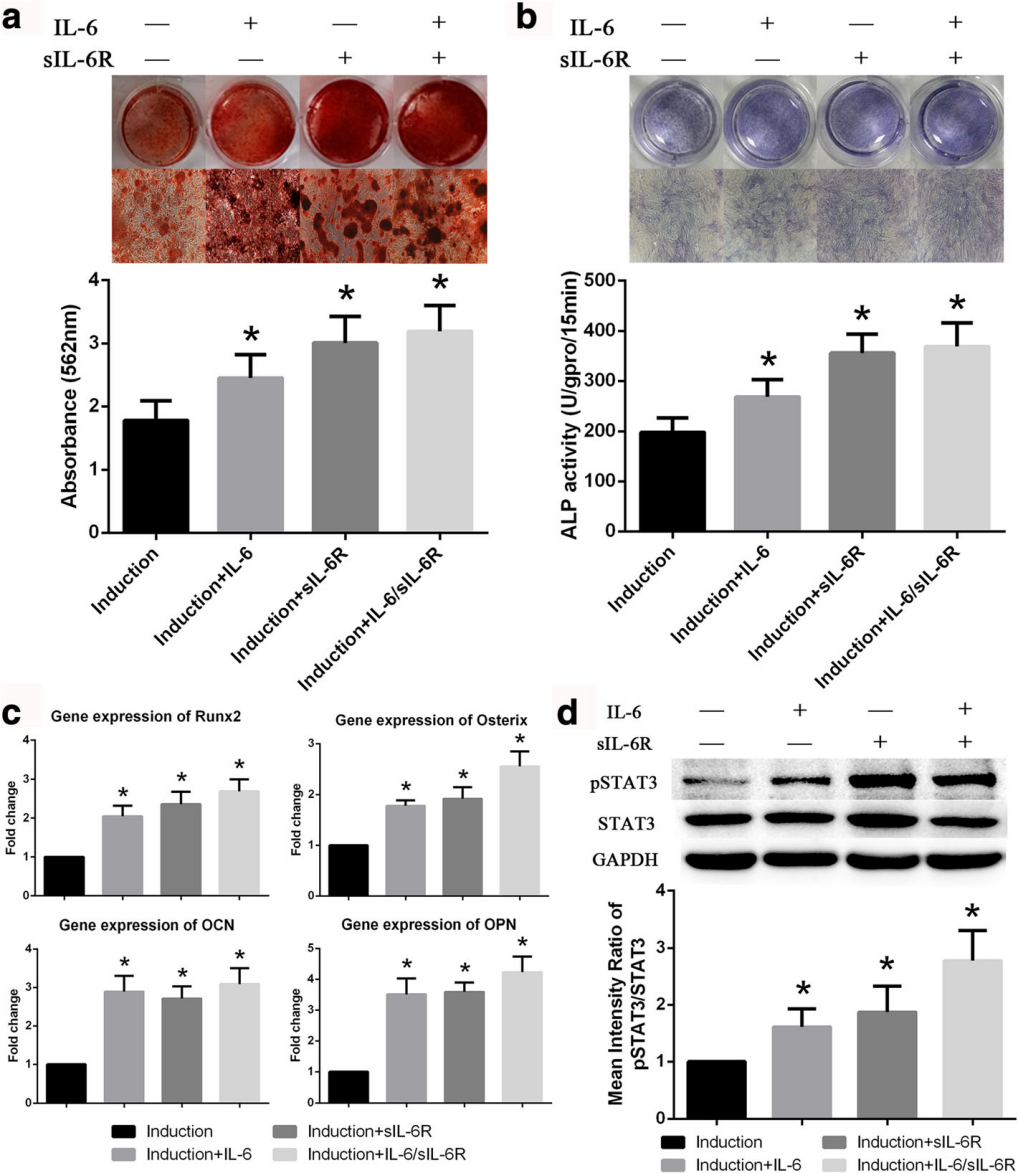
Exogenous IL-6 and sIL-6R promote osteogenic differentiation in BM-MSCs. **a** Extent of ARS staining in BM-MSCs was clearly increased by exogenous IL-6, sIL-6R or both. **b** ALP staining and activity of BM-MSCs were noticeably increased by exogenous IL-6 and sIL-6R. **c** Expression of osteoblastic marker genes in BM-MSCs was also promoted by exogenous IL-6 and sIL-6R. **d** STAT3 phosphorylation was markedly increased in response to stimulation with exogenous IL-6 and sIL-6R. Data are presented as the means ± SD of 15 samples per group. *Indicates *P* < 0.05 compared to the induction group. *IL-6* interleukin 6, *IL-6R* interleukin 6 receptor,

Exogenous IL-6 and sIL-6R were added to confirm the respective roles of IL-6 and IL-6R in the osteogenic differentiation of BM-MSCs. As shown in Fig. 4a, exogenous IL-6 and sIL-6R, both alone and in combination, increased the extent of ARS staining and positive cells. ALP staining also demonstrated similar results (Fig. 4b). Consistent with the results of ARS and ALP staining, the expression of osteoblastic marker genes was also increased in response to exogenous IL-6 and sIL-6R (Fig. 4c). Additionally, exogenous IL-6 and sIL-6R increased the phosphorylation of STAT3 during the osteogenic differentiation of BM-MSCs (Fig. 4d). Therefore, IL-6 and IL-6R promoted the osteogenic differentiation in BM-MSCs.

### Decreasing IL-6- and IL-6R expressions inhibit osteogenic differentiation in BM-MSCs

**Fig. 5:**
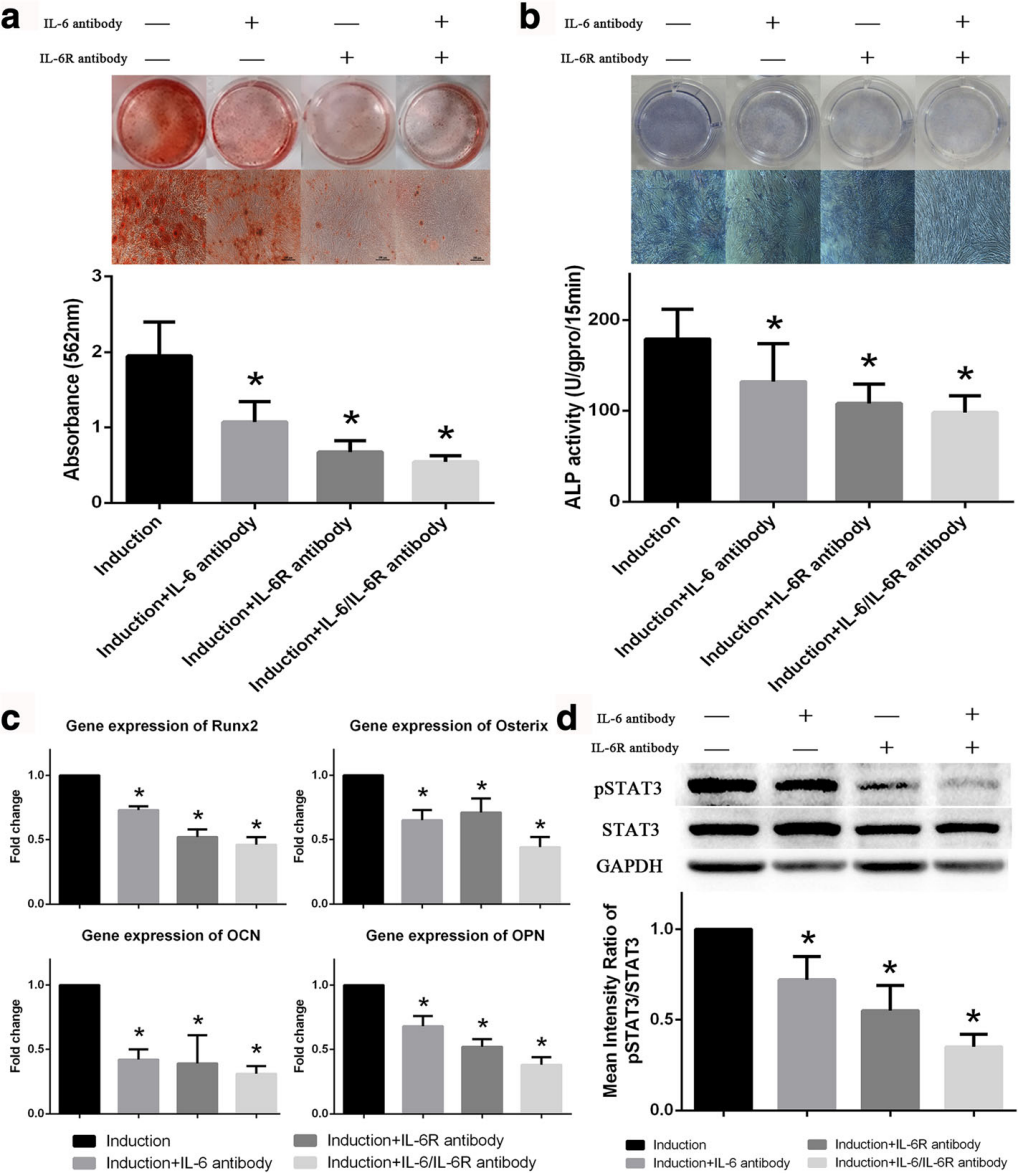
IL-6- and IL-6R-neutralizing antibodies inhibit osteogenic differentiation in BM-MSCs. **a** IL-6, sIL-6R or both inhibited the extent of ARS staining in BM-MSCs. **b** ALP staining and activity of BM-MSCs were reduced by IL-6- and IL-6R-neutralizing antibodies. **c** Expression of osteoblastic marker genes in BM-MSCs was also inhibited by IL-6- and IL-6R-neutralizing antibodies. **d** IL-6- and IL-6R-neutralizing antibodies also inhibited STAT3 phosphorylation in BM-MSCs during osteogenic differentiation. Data are presented as the means ± SD of 15 samples per group. *Indicates *P* < 0.05 compared to the induction group. *ALP* alkaline phosphatase, *IL-6* interleukin 6, *IL-6R* interleukin 6 receptor

IL-6- and IL-6R-neutralizing antibodies were used to further confirm the roles of IL-6/IL-6R in the osteogenic differentiation of BM-MSCs. ARS and ALP staining results demonstrated that IL-6- and IL-6R-neutralizing antibodies, both alone and in combination, reduced the osteogenic differentiation potential of BM-MSCs (Fig. 5a, b). Similar results were obtained regarding the gene expression of Runx2, Osterix, OCN and OPN detected by qRT-PCR (Fig. 5c). In addition, the phosphorylation of STAT3 was reduced by IL-6- and IL-6R-neutralizing antibodies (Fig. 5d). Besides, we found that the osteogenic differentiation abilities of BM-MSCs were obviously inhibited after transfected by Lv-IL6 and Lv-IL6R, which was consistent with the results of neutralizing antibody assays (Additional file 2: Figure S1). These results further confirmed the role of IL-6 and IL-6R in promoting the osteogenic differentiation of BM-MSCs.

### IL-6 and IL-6R stimulate each other’s expression in BM-MSCs

**Fig. 6:**
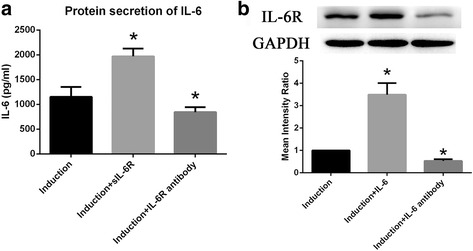
IL-6 and IL-6R stimulate each other’s expression in BM-MSCs. **a** IL-6 secretion in BM-MSCs was increased by exogenous sIL-6R but decreased by IL-6R-neutralizing antibody during osteogenic differentiation. **b** Western blotting results showed that mIL-6R expression in BM-MSCs was induced by exogenous IL-6 but reduced by IL-6-neutralizing antibody. Data are presented as the means ± SD of 15 samples per group. *Indicates *P* < 0.05 compared to the induction group. *IL-6* interleukin 6, *IL-6R* interleukin 6 receptor

We also studied whether IL-6 or IL-6R can affect each other’s expression. Exogenous sIL-6R increased IL-6 secretion by BM-MSCs during osteogenic differentiation, but IL-6R-neutralizing antibody exerted the opposite effect (Fig. 6a). Similarly, exogenous IL-6 also increased IL-6R expression in BM-MSCs, and IL-6-neutralizing antibody exerted the opposite effect (Fig. 6b).

## Discussion

In this study, we observed increased IL-6 secretion and mIL-6R expression during osteogenic differentiation in BM-MSCs. In response to stimulation with exogenous IL-6, IL-6R or both, ARS and ALP staining, osteoblastic gene expression and STAT3 phosphorylation were increased. In addition, treatment with IL-6- or IL-6R-neutralizing antibodies exerted opposite effects. Moreover, we observed a positive feedback loop between IL-6 and IL-6R, through which these two factors stimulated each other’s expression in BM-MSCs. Therefore, IL-6 and mIL-6R form complexes to activate the downstream STAT3 signaling pathway, which subsequently promotes osteogenic differentiation in BM-MSCs in an autocrine/paracrine manner.

IL-6 is an important cytokine that is involved in various physiological and pathological processes [5]. Specifically, IL-6 plays an important role in maintaining the dynamic equilibrium between bone formation and resorption [12]. A previous study has reported that IL-6 secreted by osteoblasts promotes the activation and differentiation of osteoclasts [13]. MSCs are the predominant cell of origin of osteoblasts in vivo, and these cells both secrete and respond to IL-6 [6, 14]. Kondo et al*.* have demonstrated that although IL-6 expression decreases during differentiation, IL-6 can accelerate chondrogenic differentiation in MSCs [7]. Huh et al*.* also demonstrated that in response to Toll-like receptor activation, IL-6 secretion from adipose MSCs can increase, and this promotes the osteogenic differentiation of adipose-derived MSCs [15]. In this study, we found that the level of IL-6 secreted by BM-MSCs increased during osteogenic differentiation and activated the STAT3 signaling pathway, which further facilitated osteogenic differentiation in BM-MSCs. These results were mostly consistent with previous reports. However, several differences between the results of other studies and ours should be mentioned. On one hand, we found that BM-MSCs could continually secrete IL-6 during osteogenic differentiation without requiring other stimuli. On the other hand, IL-6 secreted by BM-MSCs gradually increases, rather than decreases, with the progression of osteogenic differentiation. These results suggest that during osteogenic differentiation of BM-MSCs, IL-6 acts as an autocrine/paracrine feedback loop to promote differentiation. We speculate that these differences may arise due to differences in the tissue source of MSCs.

Both mIL-6R and sIL-6R bind to IL-6 and phosphorylate STAT3 to regulate various cellular functions [8]. As previously reported, IL-6R, together with IL-6, participates in bone metabolism in vivo [12]. In our study, BM-MSCs expressed mIL-6R rather than sIL-6R during osteogenic differentiation, which further promoted osteogenic differentiation in BM-MSCs through the STAT3 signaling pathway. However, whether MSCs can express both forms of IL-6R is still controversial. Some studies have claimed that IL-6R is not expressed on the membrane of MSCs and that IL-6 alone cannot promote osteogenic differentiation in MSCs without exogenous sIL-6R [15, 16]. In contrast, recent studies have demonstrated that IL-6R expression is not only detected in MSCs but also gradually increased during differentiation [7]. Moreover, IL-6R levels in MSCs have been associated with disease prognosis [17]. It has been reported that IL-6R expression is regulated by various endogenous and exogenous factors, including dexamethasone, which was used in our study [18, 19]. Therefore, we conclude that controversies regarding IL-6R expression may have resulted from differences in experimental conditions in vitro and differentiation environments in vivo.

Because both IL-6 and IL-6R form complexes to regulate the function of various cells that participate in many physiological and pathological processes, these two molecules are indispensable for bodily functions [5]. IL-6 and IL-6R are known to tightly regulate each other’s expression to establish a dynamic equilibrium [19]. For example, IL-6 can stimulate IL-6R expression in hepatocytes and bronchial epithelial cells, which is indicative of a positive feedback loop in these cells [20, 21]. Nevertheless, the regulatory relationship between IL-6 and IL-6R in MSCs remains unclear. To elucidate the relevant mechanism, in this study, we showed a positive correlation between IL-6 and IL-6R levels because the expression of both proteins was upregulated/downregulated via relative exogenous proteins/neutralizing antibodies. In addition, IL-6 and IL-6R were found to stimulate each other’s expression in BM-MSCs during osteogenic differentiation. Therefore, IL-6 and IL-6R form complex to promote osteogenic differentiation of BM-MSCs as an autocrine/paracrine positive feedback loop. However, definitive regulatory mechanisms need to be addressed in the future.

The IL-6/IL-6R/STAT3 signaling pathway has been shown to play a critical role in bone metabolism [22]. On one hand, IL-6 affects bone mass by modulating the balance between osteocytes and osteoclasts [23]. On the other hand, IL-6R is maintained at a relatively high level in the serum to modulate bone formation, and polymorphisms in the IL-6R gene are related to bone mineral density [24]. Dysregulation of these two molecules can lead to abnormal bone metabolism and have been linked with the pathogenesis of diseases [25]. Previous studies have demonstrated that abnormal differentiation of MSCs is one of the important mechanism defects in bone metabolism in diseases, but the definitive mechanism still needs to be elucidated [10, 26, 27]. In this study, we demonstrated the critical role of the IL-6/IL-6R complex in the osteogenic differentiation of BM-MSCs. We further concluded that a dysregulated IL-6/IL-6R/STAT3 signaling pathway may lead to abnormal bone metabolism under pathological conditions by affecting the differentiation of MSCs. Besides, tocilizumab, an anti-IL-6R antibody, has been widely used for various diseases and was shown to mitigate dysregulated bone metabolism [5, 28]. Our results suggest that anti-IL-6 antibodies could also have potential to be used in the clinic as a novel treatment.

However, several limitations still exist in this study, including the lack of understanding of the regulatory mechanism of IL-6 and IL-6R expression in BM-MSCs. Besides, the expressions and functions of IL-6 and IL-6R during osteogenic differentiation of MSCs in vivo are also still unclear. These limitations should be addressed in future studies.

## Conclusions

In summary, we demonstrated that the IL-6/IL-6R complex promotes osteogenic differentiation in BM-MSCs. Our findings thus enhance the understanding of the IL-6/IL-6R/STAT3 signaling pathway in bone metabolism in vivo, which may help to illuminate the pathogenesis of diseases and develop novel targeted medicines.

## Additional files

Additional file 1: **Table S1.** Primers used for qRT-PCR. Primer information for gene. (DOCX 15 kb)

Additional file 2: **Figure S1.** Lv-IL6 and Lv-IL6R inhibit osteogenic differentiation in BM-MSCs. **(A)** Lv-IL6 inhibited the IL-6 secretion of BM-MSCs. **(B)** Lv-IL6R decreased the IL-6R expression of BM-MSCs. **(C)** Lv-IL6 and Lv-IL6R both decreased the ARS quantification and staining of BM-MSCs. **(D)** Lv-IL6 and Lv-IL6R also decreased the ALP quantification and staining of BM-MSCs. Data are presented as the means ± SD of 15 samples per group. ^*^Indicates *P* < 0.05 compared to the induction group. (TIF 9539 kb)
