# Supplementary material for: Interleukin-6/interleukin-6 receptor complex promotes osteogenic differentiation of bone marrow-derived mesenchymal stem cells
Source: Stem Cell Res Ther. 2018 Jan 22;9:13. doi: 10.1186/s13287-017-0766-0 (PMC5776773; doi:10.1186/s13287-017-0766-0)
Supplement: Supplementary file 1 — Table S1. Primers used for qRT-PCR. Primer information for gene. (DOCX 15 kb) [file 13287_2017_766_MOESM1_ESM.docx]

**Additional 1: Table S1 Primers used for qRT-PCR**

| **Gene** | **Accession No.** | **Forward primer**  **(5`-3`)** | **Reverse primer**  **(5`-3`)** | **Product size(bp)** |
| --- | --- | --- | --- | --- |
| GAPDH | [NM_001256799](http://www.ncbi.nlm.nih.gov/entrez/query.fcgi?cmd=Search&db=Nucleotide&term=NM_001256799) | GGAGCGAGATCCCTCCAAAAT | GGCTGTTGTCATACTTCTCATGG | 197 |
| IL-6 | [NM_173672](http://www.ncbi.nlm.nih.gov/entrez/query.fcgi?cmd=Search&db=Nucleotide&term=NM_173672) | CCTGAACCTTCCAAAGATGGC | TTCACCAGGCAAGTCTCCTCA | 75 |
| IL-6R | [NM_181359](http://www.ncbi.nlm.nih.gov/entrez/query.fcgi?cmd=Search&db=Nucleotide&term=NM_181359) | CCCCTCAGCAATGTTGTTTGT | CTCCGGGACTGCTAACTGG | 171 |
| Runx2 | [NM_001015051](http://www.ncbi.nlm.nih.gov/entrez/query.fcgi?cmd=Search&db=Nucleotide&term=NM_001015051) | TCAACGATCTGAGATTTGTGGG | GGGGAGGATTTGTGAAGACGG | 81 |
| Osterix | [NM_152860](http://www.ncbi.nlm.nih.gov/entrez/query.fcgi?cmd=Search&db=Nucleotide&term=NM_152860) | CCTCTGCGGGACTCAACAAC | AGCCCATTAGTGCTTGTAAAGG | 128 |
| OCN | [NM_199173](http://www.ncbi.nlm.nih.gov/entrez/query.fcgi?cmd=Search&db=Nucleotide&term=NM_199173) | CACTCCTCGCCCTATTGGC | CCCTCCTGCTTGGACACAAAG | 112 |
| OPN | [NM_001251830](http://www.ncbi.nlm.nih.gov/entrez/viewer.fcgi?db=nucleotide&id=352962175) | GAAGTTTCGCAGACCTGACAT | GTATGCACCATTCAACTCCTCG | 91 |

GAPDH, glyceraldehyde-3-phosphate dehydrogenase ; OCN, osteocalcin; OPN, osteopontin.
