# Supplementary figures and images for: Interleukin-6/interleukin-6 receptor complex promotes osteogenic differentiation of bone marrow-derived mesenchymal stem cells
Source: Stem Cell Res Ther. 2018 Jan 22;9:13. doi: 10.1186/s13287-017-0766-0 (PMC5776773; doi:10.1186/s13287-017-0766-0)

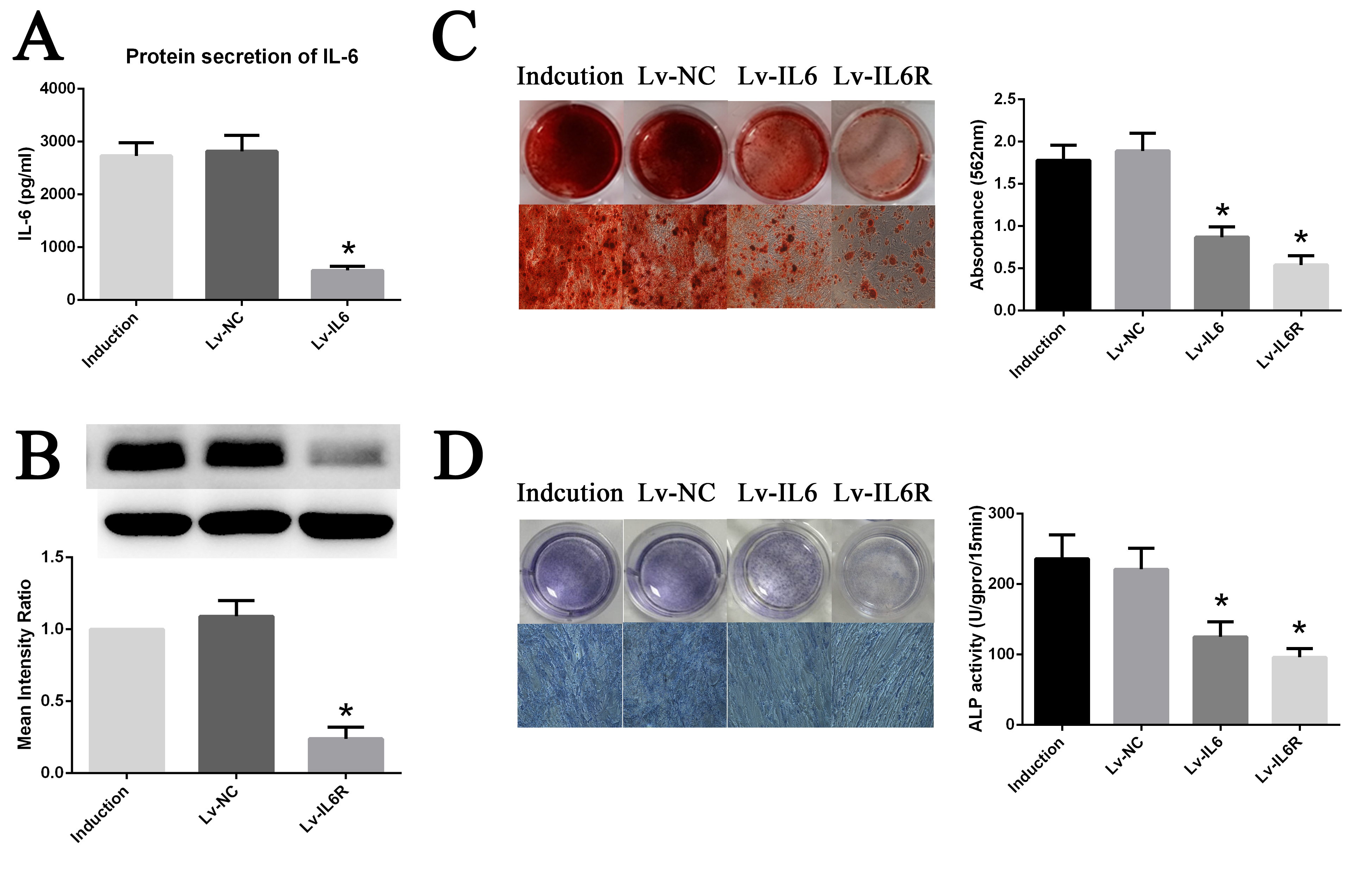

Supplement: Supplementary file 2 — Figure S1. Lv-IL6 and Lv-IL6R inhibit osteogenic differentiation in BM-MSCs. (A) Lv-IL6 inhibited the IL-6 secretion of BM-MSCs. (B) Lv-IL6R decreased the IL-6R expression of BM-MSCs. (C) Lv-IL6 and Lv-IL6R both decreased the ARS quantification and staining of BM-MSCs. (D) Lv-IL6 and Lv-IL6R also decreased the ALP quantification and staining of BM-MSCs. Data are presented as the means ± SD of 15 samples per group. *Indicates P < 0.05 compared to the induction group. (TIF 9539 kb) [file 13287_2017_766_MOESM2_ESM.tif]
